# The genome sequence of *Artibeus lituratus *(Chiroptera, Phyllostomidae, Stenodermatinae; Olfers, 1818).

**DOI:** 10.12688/wellcomeopenres.23088.1

**Published:** 2025-04-01

**Authors:** Nancy B. Simmons, Melissa R. Ingala, Brian P. O'Toole, Giulio Formenti, Philip Philge, Ning Zhang, Erich D. Jarvis, Jonathan L. Gray, Kirsty McCaffrey, Nadolina Brajuka, Myrtani Pieri, Meike Mai, Emma C. Teeling, Sonja C. Vernes, The Bat Biology Foundation, The Bat1K Consortium

**Affiliations:** 1 Department of Mammalogy, Division of Vertebrate Zoology,, American Museum of Natural History, New York, NY 10024, USA; 2 Department of Biological Sciences, Fairleigh Dickinson University, Madison, New Jersey, NJ 07940, USA; 3 Division of Mammals, Department of Vertebrate Zoology, National Museum of Natural History, Smithsonian Institution, Washington, District of Columbia, Washington, DC 20560, USA; 4 Paratus Sciences, New York, USA; 5 Vertebrate Genome Lab, The Rockefeller University, New York, USA; 6 Department of Life Sciences, School of Life and Health Sciences,, University of Nicosia, NIcosia, 2417, Cyprus; 7 School of Biology, University of St Andrews, St Andrews, Scotland, UK; 8 University College Dublin School of Biology and Environmental Science, Dublin, Leinster, Ireland; 9 Wellcome Sanger Institute, Wellcome Genome Campus, Cambridgeshire, CB10 1SA, UK

**Keywords:** Artibeus lituratus, genome sequence, chromosomal, Bat1K

## Abstract

We present a genome assembly from an individual male *Artibeus lituratus* (Chordata; Mammalia; Chiroptera; Phyllostomidae). The genome sequence is 2.15 in span. The majority of the assembly is scaffolded into 30 chromosomal pseudomolecules, with the X and Y sex chromosomes assembled.

## Species taxonomy

Eukaryota; Metazoa; Chordata; Craniata; Vertebrata; Euteleostomi; Mammalia; Eutheria; Laurasiatheria; Chiroptera; Yangochiroptera; Phyllostomidae; Stenodermatinae; Stenodermatini; Artibeina; *Artibeus*; *Artibeus lituratus* ( Baker *et al.*, 2016; Cirranello *et al.*, 2016; Simmons & Cirranello, 2024).

## Introduction

**Figure 1.**
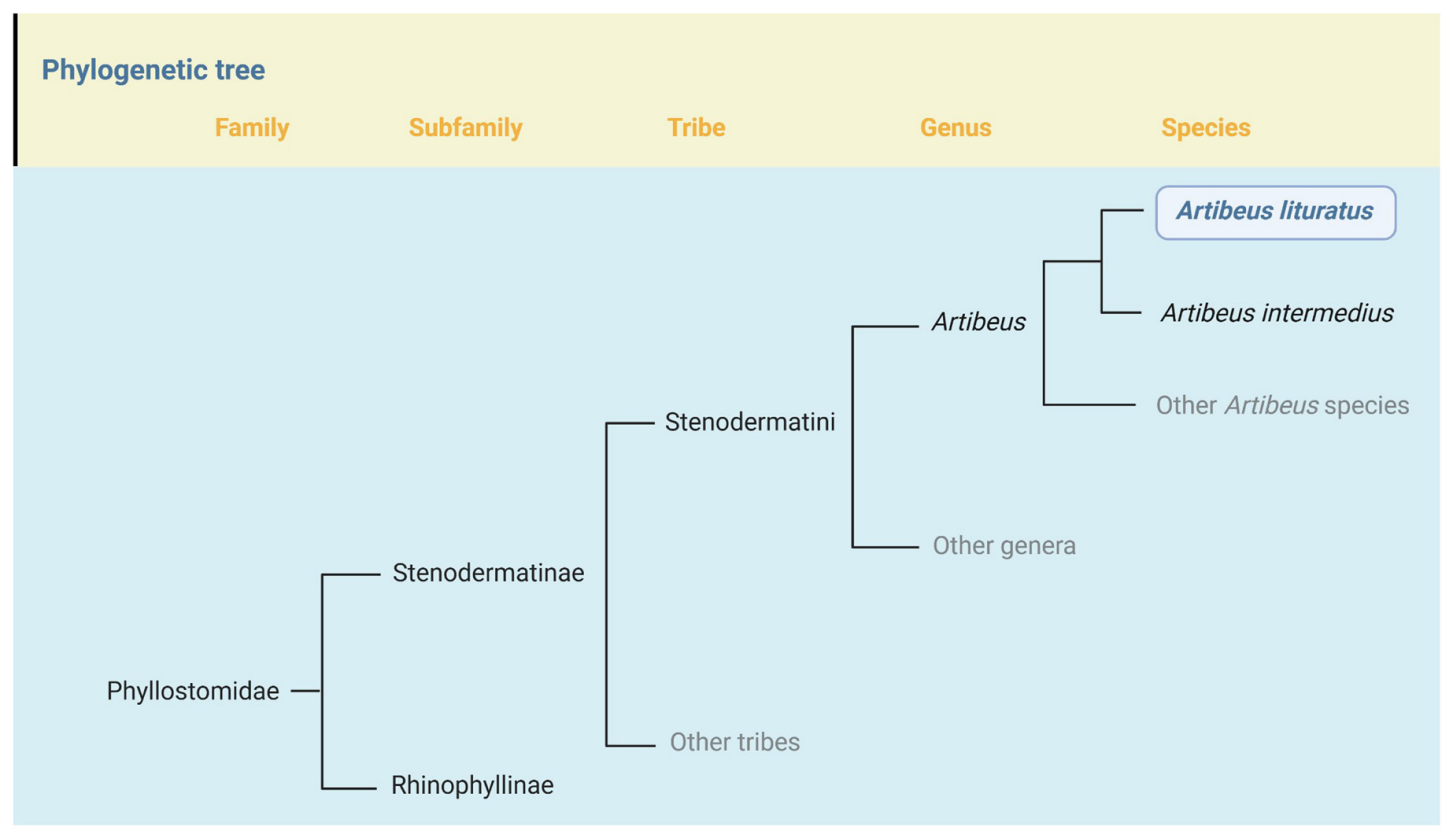
Position of *Artibeus lituratus* in the phylogeny of Family Phyllostomidae. *Artibeus lituratus* is one of 13 species currently recognized in the genus *Artibeus* ( Simmons & Cirranello, 2024). *Artibeus* belongs to the Tribe Stenodermatini in the Subfamily Stenodermatinae, which currently includes 19 genera and 77 species ( Baker *et al.*, 2016; Cirranello *et al.*, 2016; Simmons & Cirranello, 2024). Within *Artibeus*, the closest relative of *A. literatus* is *A. intermedius*; until recently these taxa were considered conspecific. Phylogeny based on Botero-Castro *et al.* (2013), Larsen *et al.* (2013), and Rojas *et al.* (2016).

The genus *Artibeus* is a clade of relatively large-bodied frugivorous bats belonging to the Tribe Stenodermatini within the Subfamily Stenodermatinae ( Figure 1) ( Baker *et al.*, 2016; Cirranello *et al.*, 2016; Simmons & Cirranello, 2024). *Artibeus* are generally believed to be fig specialists although they also consume a wide variety of other fruits ( de Souza Laurindo & Vinzentin-Bugoni, 2020; Emmons & Feer, 1997; Ingala *et al.*, 2021; Reid, 2009). *Artibeus* bats are common throughout the Neotropics with multiple species often occurring in sympatry ( Emmons & Feer, 1997; Larsen *et al.*, 2013; Marques-Aguiar, 1994; Marques-Aguiar, 2007; Reid, 2009). *Artibeus lituratus* (Olfers, 1818), the largest species of *Artibeus*, is found from central Mexico south through most of Central America including Guatemala, Belize, Honduras, Nicaragua, Costa Rica, Panama, and possibly El Salvador ( Larsen *et al.*, 2013; Mammal Diversity Database, 2024). In South America, *A. lituratus* occurs in Colombia, Venezuela, Guyana, Suriname, French Guiana, Ecuador, Peru, Bolivia, Brazil, Paraguay, and Argentina, and it also occurs in the Caribbean on Trinidad and Tobago, Saint Vincent and the Grenadines, Grenada, Barbados, Martinique, and Saint Lucia ( Larsen *et al.*, 2013; Mammal Diversity Database, 2024).

Several subspecies have been recognized within *Artibeus lituratus* including the nominal subspecies *Artibeus l. lituratus* (Olfers, 1818), *A. l. koopmani* Wilson, 1991, and *A. l. palmarum* Allen and Chapman, 1897, and it is likely that this complex includes cryptic species diversity ( Larsen *et al.*, 2010; Larsen *et al.*, 2013; Simmons, 2005). Some authors have indicated that *Artibeus intermedius* Allen, 1897 should be considered a junior synonym of *A. lituratus* (e.g., Guerrero *et al.*, 2008; Hoofer *et al.*, 2008; Lim *et al.*, 2004; Marques-Aguiar, 1994; Redondo *et al.*, 2008; Simmons, 2005) but others have concluded that these are different species (e.g., Davis, 1984; Koopman, 1994; Larsen *et al.*, 2013; Marchán-Rivadeneira *et al.*, 2012; Reid, 2009; Wilson, 1991). Recent comprehensive bat classifications (e.g., Mammal Diversity Database, 2024; Simmons & Cirranello, 2024) follow Larsen *et al.* (2013) in treating *A intermedius* as a species distinct from *A. lituratus*. Both species occur in sympatry at the site in Belize where the individual reported below was captured (see Methods).

**Figure 2.**
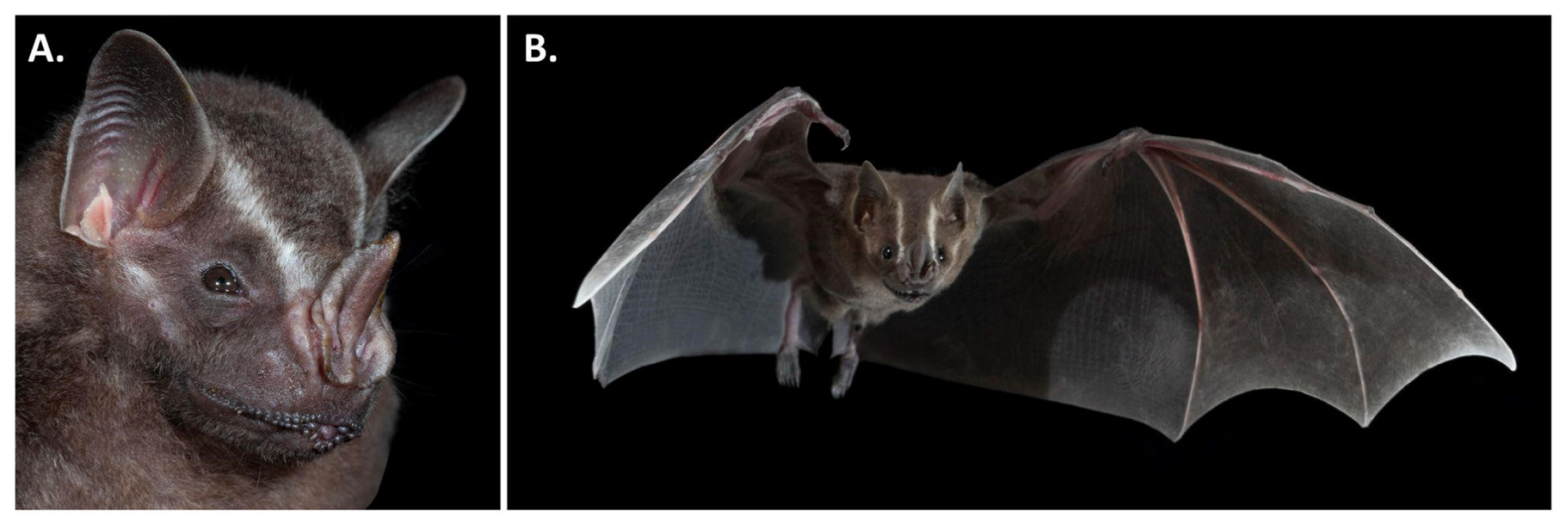
*Artibeus lituratus.* Adult individuals of *Artibeus lituratus*. **A**, portrait showing facial striping, chin, and noseleaf morphology. **B**, *Artibeus lituratus* in flight [Photos taken at Lamanai, Belize by Brock and Sherri Fenton].

*Artibeus lituratus* ( Figure 2) can be distinguished from congeners on the basis of a series of morphological traits including its very large size (forearm 68-78 mm, greatest length of skull 28-31 mm, 50-86 g), prominent and well-defined white facial stripes, brown dorsal fur and brown wings, ventral fur that is not conspicuously frosted with white, uropatagium and legs that are well furred, preorbital and postorbital processes well developed, M1 not triangular in occlusal view, M1 lacking a well-developed hypocone, and M3 absent but m3 present ( Emmons & Feer, 1997; Koopman, 1994; Marquez-Aguiar, 2007; Reid, 2009).

*Artibeus lituratus* is common to abundant throughout much of its range, especially in lowland evergreen and deciduous forests, although it may be uncommon or absent in very dry or highly disturbed areas ( Marques-Aguiar, 2007; Reid, 2009). It is sometimes found in urban environments where fruit trees are common ( Bobrowiec & Cunha 2010; de Souza Laurindo & Vizentin-Bugoni, 2020). This species roosts in caves, tunnels, hollow trees, recesses under branches, vine tangles, and foliage, and it apparently prefers roosts 3-30 m above the ground ( Morrison, 1980; Reid, 2009; Simmons & Voss, 1998). Groups seem to typically be composed of a single male and several females ( Morrison, 1980; Reid, 2009). The diet of *A. lituratus* variously consists of figs ( *Ficus*), fruits of *Dipteryx*, *Piper*, *Cecropia*, *Solanum*, *Vismia*, and other trees and shrubs, and flowers and pollen of various canopy trees, although they also occasionally eat insects and leaves ( Bobrowiec & Cunha, 2010; de Souza Laurindo & Vizentin-Bugoni, 2020; Ingala *et al.*, 2021; Marquez-Aguiar, 2007; Morrison, 1980; Reid, 2009; Zortea & Mendes, 1993). These bats are important seed dispersers for at least ten species of rainforest trees ( dos Reis & Peracchi, 1987).

*Artibeus lituratus* is classified as Least Concern in the IUCN Redlist of Threatened Species because of its wide distribution, presumed large population size, and because it is unlikely to be declining at the rate required to qualify for listing in a threatened category ( Barquez *et al.*, 2015).

## Genome sequence report

The genome was sequenced from a single male *Artibeus lituratus* (field number BZ-128, catalog number AMNH:Mammalogy:280692 collected at the Lamanai Archaeological Reserve, Orange Walk District, Belize, on 9 November 2021. A total of 48-fold coverage in Pacific Biosciences Hi-Fi long reads (contig N50 [70 Mb) was generated after removal of all reads shorter than 10kb. Primary assembly contigs were scaffolded with chromosome confirmation Hi-C data. The final assembly has a total length of 2.15 Gb in 535 sequence scaffolds with a scaffold N50 of 160 Mb. The majority, 97.7%, of the assembly sequence was assigned to 30 chromosomal-level scaffolds, representing 14 autosomes (numbered by sequence length, and the X sex chromosome). The assembly has a BUSCO ( Simao *et al.*, 2015) completeness of 98.2% using the laurasiatheria reference set. Chromosomal pseudomolecules in the genome assembly of *Artibeus literatus* are shown in Table 2.

## Methods

The *Artibeus lituratus* specimen was a male individual collected on an American Museum of Natural History (AMNH) field expedition at the Lamanai Archaeological Reserve in the Orange Walk District of Belize. The individual sampled was identified as *A. lituratus* based on morphometrics (e.g., forearm length, body mass) and morphological traits (e.g., brightness of eye stripes, fur color and distribution) as described above. The bat was caught in a ground-level mist net set near the Stela Temple in the Lamanai Archaeological Reserve (17.76639 N, 88.65225 W). All efforts were made to minimize any distress or suffering by the animal. The individual sampled was subjected to minimal handling after capture, and it was held in a clean cloth bag after capture as per best practices for field containment of bats. After species identification, the individual was euthanized humanely the same night it was captured. The animal was euthanized by isoflurane inhalation (<1 ml to moisten cotton ball), an approved and humane euthanasia method that rapidly causes unconsciousness and eventually death. Bats euthanized by this method are rendered unconscious within seconds due to their high respiration rate, and death occurs within a minute or two with no significant suffering by the animal. Capture and sampling were conducted under Belize Forest Department Permit FD/WL/1/21(16) and Belize Institute of Archaeology Permit IA/S/5/6/21(01), and samples were exported under Belize Forest Department permit FD/WL/7/22(07). All work was conducted with approval by the AMNH Institutional Animal Care and Use Committee (AMNHIACUC-20210614). All data were recorded and reported in accordance with the ARRIVE guidelines ( Kilkenny *et al.*, 2010) – see data availability section and Table 1. Tissues were removed from the subject individual immediately following euthanasia and were flash-frozen in a liquid nitrogen dry shipper, with the cold chain maintained from field to museum to laboratory.

**Table 1.** Genome data for *Artibeus lituratus*.

*Project accession data*	
Assembly identifier	GCA_038363095.2
Species	***Artibeus lituratus***
Specimen	mArtLit1
NCBI taxonomy ID	94809
BioProject	Bat1K: Accession: PRJNA489245; ID: 489245
BioSample ID	SAMN40002247
Isolate information	Male - muscle
Span (Gb)	2.15
Number of contigs	561
Contig N50 length (Mb)	70
Number of scaffolds	535
Scaffold N50 length (Mb)	160
Longest scaffold (Mb)	2.141

* BUSCO scores based on the laurasiatheria_odb10 BUSCO set using v5.0.0. C= complete [S= single copy, D=duplicated], F=fragmented, M=missing, n=number of orthologues in comparison. * *Artibeus lituratus* BUSCO scores based on laurasiatheria_odb10 BUSCO set v5.3.2.

**Table 2.** Chromosomal pseudomolecules in the genome assembly of *Artibeus lituratus*.

ENA accession	Chromosome	Size (Mb)	GC%
SUPER_1	1	245.58	0.4046
SUPER_2	2	217.35	0.4025
SUPER_3	3	193.24	0.4198
SUPER_4	4	180.62	0.4272
SUPER_5	5	175.94	0.418
SUPER_6	6	160.30	0.4331
SUPER_7	7	150.44	0.4131
SUPER_8	8	148.99	0.4126
SUPER_9	9	129.79	0.4363
SUPER_10	10	120.93	0.4486
SUPER_11	11	110.56	0.4268
SUPER_X	X	110.17	0.3998
SUPER_12	12	99.24	0.3993
SUPER_Y	Y	60.61	0.4441
SUPER_13	13	60.57	0.465
SUPER_14	14	56.80	0.4552
SUPER_Y2	Y2	37.29	0.4552

ENA accession Chromosome Size (Mb) GC%. The chromosome number of *Artibeus lituratus* is 2n=30.

DNA was extracted using Nanobind extraction from muscle tissue following the Circulomics Nanobind HMW DNA Extraction Protocol. Pacific Biosciences HiFi libraries were constructed according to the manufacturer's instructions. Hi-C data was generated using the Arima Hi-C+ High Coverage kit from the same muscle tissue sample. Sequencing was performed by the Genomic Operations DNA Pipelines at Paratus Sciences on Pacific Biosciences Sequel IIe (HiFi reads) and Illumina NextSeq 2000 (Hi-C) instruments.

**Figure 3.**
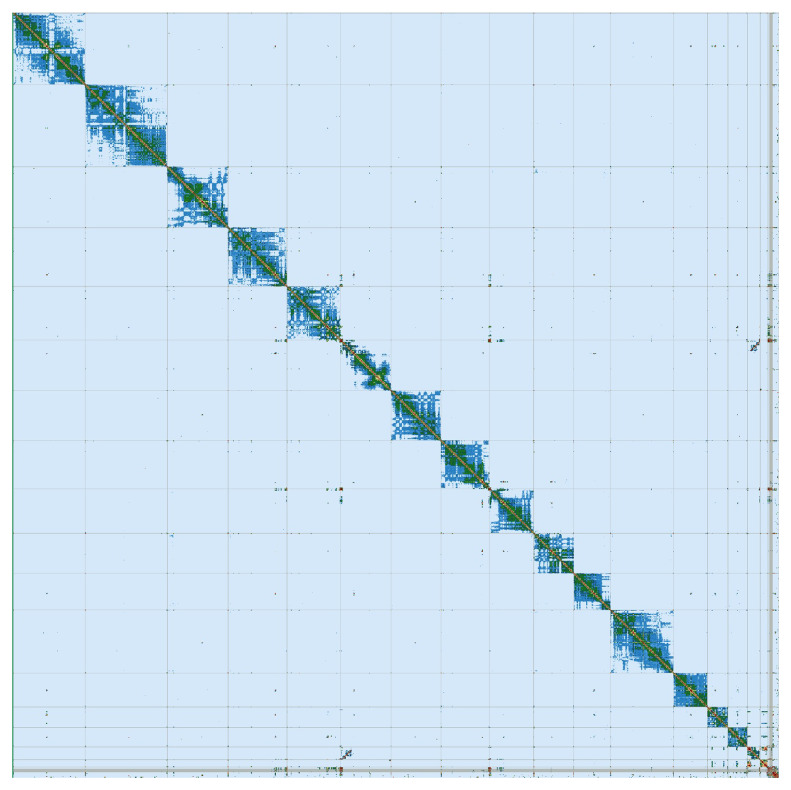
Hi-C Contact Map of the Artibeus lituratus assembly with 15 chromosomes, visualized using PretextView.

**Figure 4.**
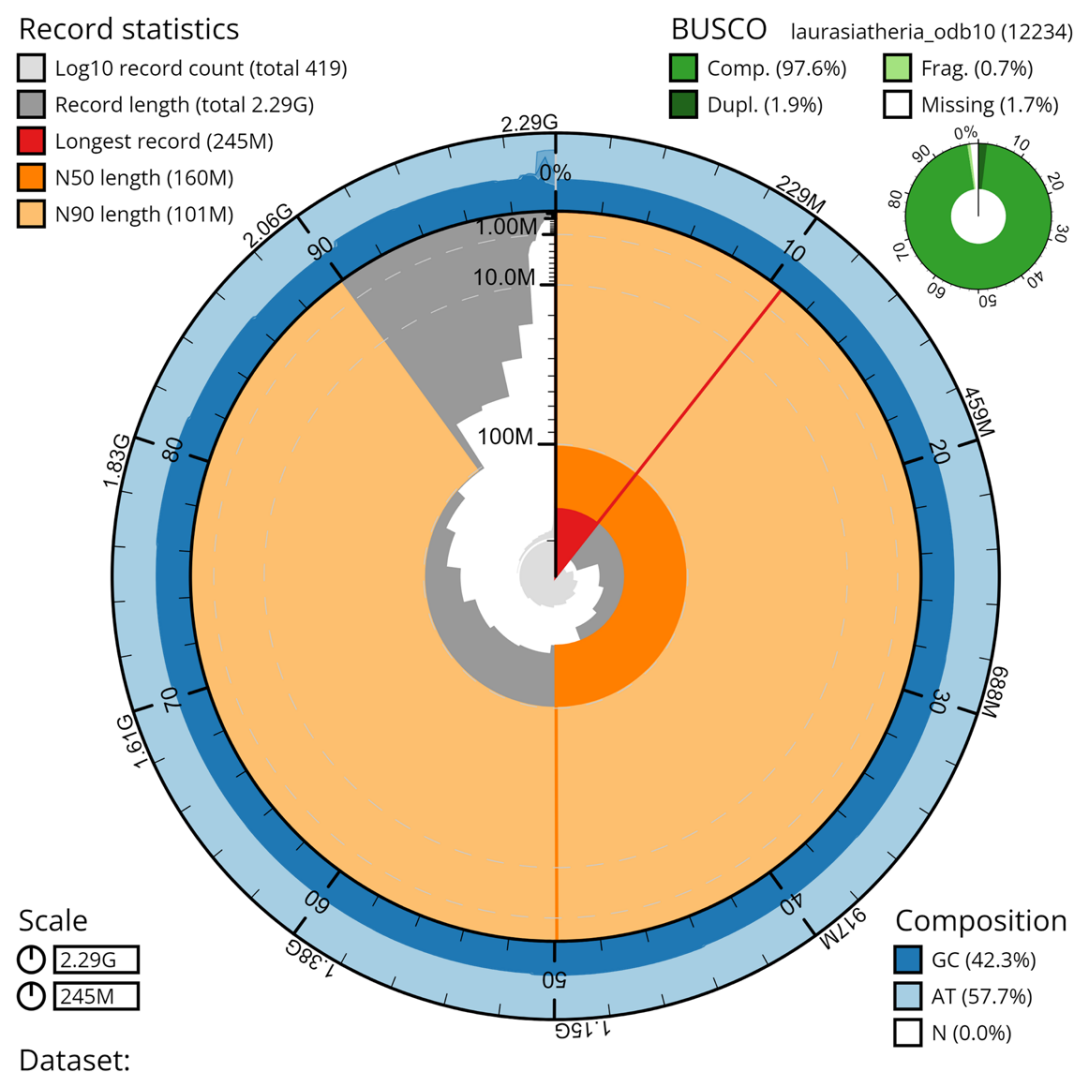
Genome assembly metrics generated using blobtoolkit for the *Artibeus lituratus* genome assembly. The larger snail plot depicts scaffold statistics including N50 length (bright orange) and base composition (blue). The smaller plot shows BUSCO completeness in green.

Assembly was carried out following the Vertebrate Genome Project Galaxy pipeline v2.0 ( Lariviere *et al.*, 2024). A brief synopsis of the method is as follows: Genome size was estimated using GenomeScope2 ( Vurture *et al.*, 2017). Hifiasm with Hi-C phasing was used for genome assembly ( Cheng *et al.*, 2021). The quality of the assembly was evaluated using Merqury ( Nurk *et al.*, 2020) and BUSCO ( Manni *et al.*, 2021). Scaffolding with Hi-C data ( Rao *et al.*, 2014) was carried out with YaHS ( Zhou *et al.*, 2023). PretextView was implemented to generate a Hi-C contact map ( Figure 3). Figure 4 – Figure 6 were generated using BlobToolKit ( Challis *et al.*, 2020). Software utilised for the *A. lituratus* analysis are depicted in Table 3.

**Figure 5.**
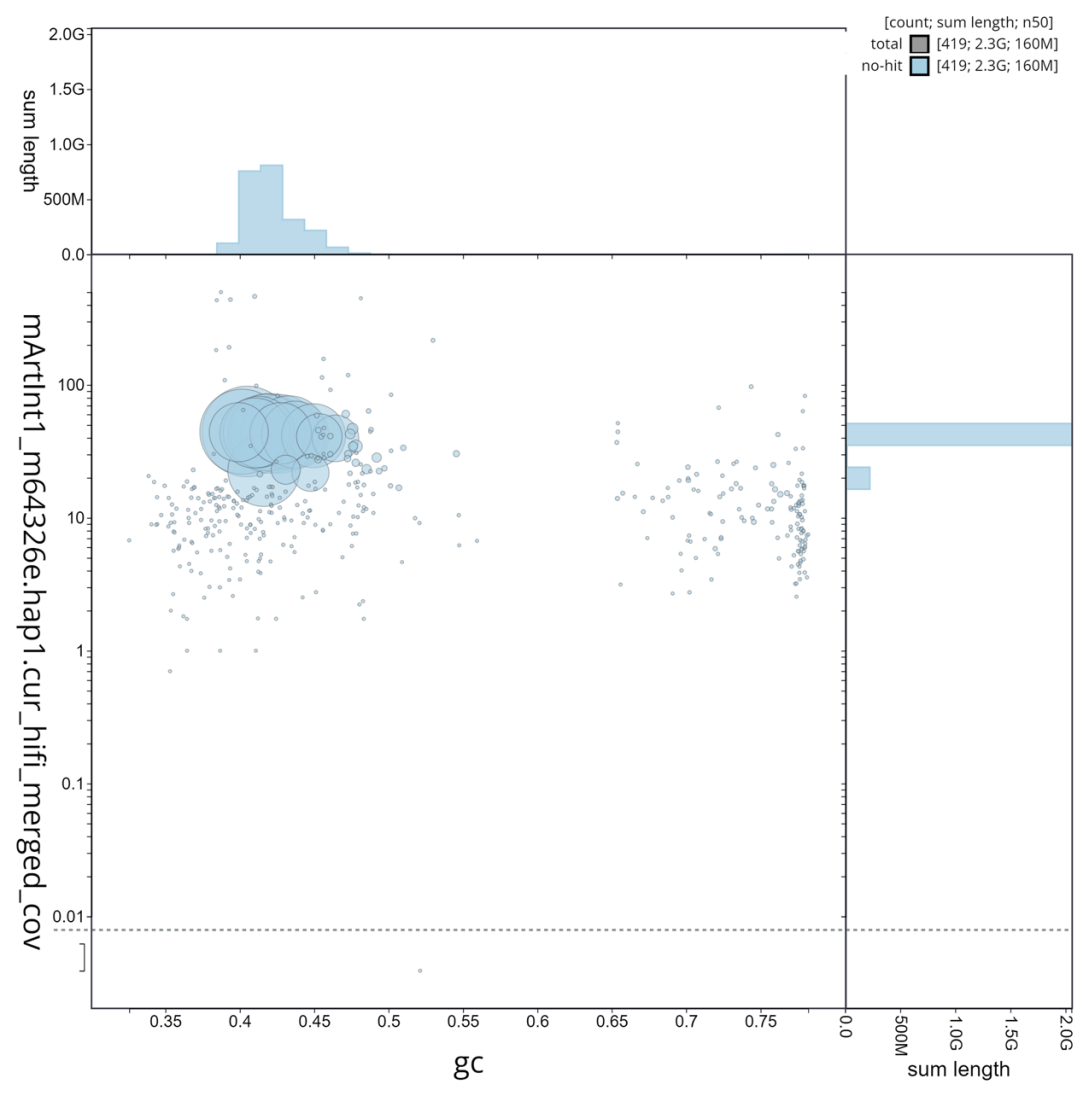
GC coverage plot generated for the *Artibeus lituratus* assembly using blobtoolkit. Individual chromosomes and scaffolds are represented by each circle. The circles are sized in proportion to chromosome/scaffold length. Histograms show the sum length of chromosome/scaffold size along each axis. Color of circles indicate taxonomic hits of each phylum represented in the assembly.

**Figure 6.**
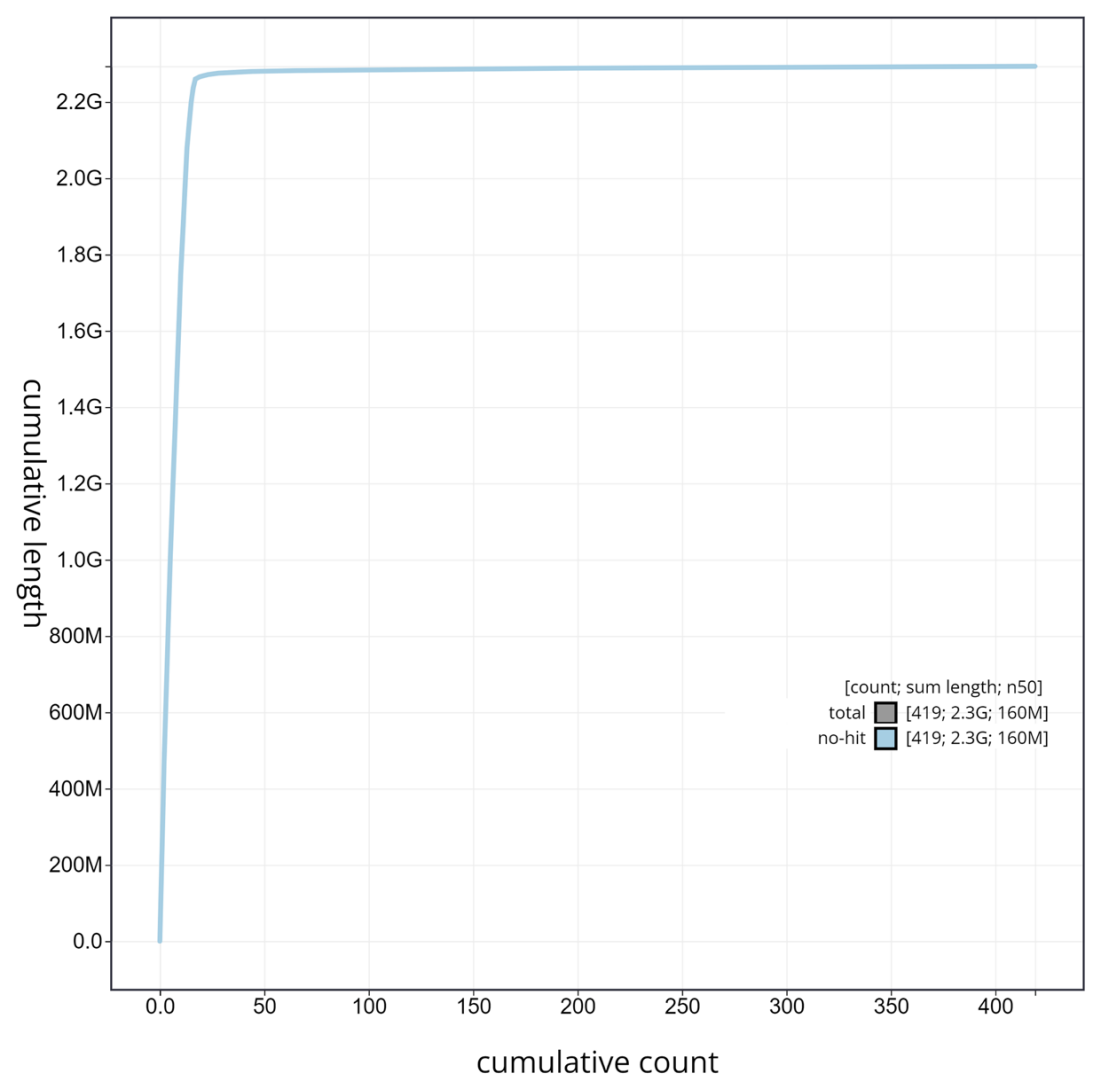
Cumulative sequence plot generated for the *Artibeus lituratus* assembly using blobtoolkit. The grey line shows the cumulative length for all chromosomes/scaffolds in the assembly. Colored lines represent phylum represented in the assembly.

**Table 3.** Software tools used.

Software tool	Version	Source
bamUtil	1.0.15	https://genome.sph.umich.edu/wiki/BamUtil:_bam2FastQ
MultiQC	1.13	https://github.com/ewels/MultiQC
Genomescope	2.0	https://github.com/tbenavi1/genomescope2.0
hifiasm	0.19.3	https://github.com/chhylp123/hifiasm
purge_dups	1.2.6	https://github.com/dfguan/purge_dups
BUSCO	5.3.2	https://busco.ezlab.org/
Merqury	1.3	https://github.com/marbl/merqury
Assembly-stats	17.02	https://github.com/rjchallis/assembly-stats
Arima-HiC Mapping Pipeline	-	https://github.com/ArimaGenomics/mapping_pipeline
YaHS	1.1	https://github.com/c-zhou/yahs
HiGlass	1.11.7	https://github.com/higlass/higlass
samtools	1.9	https://www.htslib.org/
PretextView	-	https://github.com/sanger-tol/PretextView/tree/master
BUSCO	5.7.0	https://busco.ezlab.org/
BlobToolKit	4.3.5	https://github.com/blobtoolkit/blobtoolkit
pbmm2	1.13.1	https://github.com/PacificBiosciences/pbmm2
Blast	2.15.0+	https://blast.ncbi.nlm.nih.gov/Blast.cgi

## Ethics statement

All work was conducted with approval by the AMNH Institutional Animal Care and Use Committee (AMNHIACUC-20210614). All efforts were made to minimize any distress or suffering by the animal.

## Data Availability

The *Artibeus lituratus* genome sequencing initiative is part of the Bat1K genome sequencing project. The genome assembly is released openly for reuse. Underlining data may be available for non-commercial research purposes upon request. Please email info@batbio.org for more information. All raw sequence data and the genome assembly can be found in the European Nucleotide Archive: *Artibeus lituratus* (Great Fruit-eating Bat). Accession number: GCA_038363095.2, https://www.ebi.ac.uk/ena/browser/view/GCA_038363095.2 ( BAT1K, 2024b). The raw sequence data and assembly can also be found in the NCBI database, the BioProject for *Artibeus lituratus* isolate: mArtLit1 (Great Fruit-eating Bat) is listed under Accession number: PRJNA1080658, https://www.ncbi.nlm.nih.gov/bioproject/PRJNA1080658. BAT1K, 2024a). This project is part of the broader Bat1K BioProject PRJNA489245 ( BAT1K, 2024a). Data accession identifiers are SAMN40002247 and Data accession identifiers are reported in Table 1. Zenodo: ARRIVE Checklist for "The Genome Sequence of *Artibeus lituratus* (Chiroptera, Phyllostomidae, Stenodermatinae; Olfers, 1818)". https://doi.org/10.5281/zenodo.14172686. ( BAT1K, 2024c). License: Data are available under the terms of the Creative Commons Attribution 4.0 International license (CC-BY 4.0)

## References

[ref-1] Allen JA Chapman FM : On mammals from Yucatan, with descriptions of new species. *Bulletin of the American Museum of Natural History.* 1897;9(13):247–258. Reference Source

[ref-2] Baker RJ Solari S Cirranello A : Higher level classification of phyllostomid bats with a summary of DNA synapomorphies. *Acta Chiropterologica.* 2016;18(1):1–38. 10.3161/15081109ACC2016.18.1.001

[ref-3] Barquez R Perez S Miller B : Artibeus lituratus. The IUCN Red List of Threatened Species.2015;2015: e.T2136A21995720; [Accessed 31 Aug. 2024]. 10.2305/IUCN.UK.2015-4.RLTS.T2136A21995720.en

[ref-4] BAT1K: Artibeus lituratus isolate: mArtLit1 (Great Fruit-eating Bat). NCBI BioProject, [Dataset].2024a. https://www.ncbi.nlm.nih.gov/bioproject/PRJNA1080658

[ref-5] BAT1K: Artibeus lituratus (Great Fruit-eating Bat). European Nucleotide Archive, [Dataset].2024b. https://www.ebi.ac.uk/ena/browser/view/GCA_038363095.2

[ref-36] BAT1K: ARRIVE checklist for Artibeus lituratus genome study. Zenodo.2024c. 10.5281/zenodo.14172686

[ref-6] Bobrowiec PED Cunha RM : Leaf-consuming behavior in the big fruit-eating bat, *Artibeus lituratus* (Olfers, 1818) (Chiroptera: Phyllostomidae), in an urban area of Southeastern Brazil. *Chiroptera Neotropical.* 2010;16(1):595–599. Reference Source

[ref-7] Botero-Castro F Tilak MK Justy F : Next-generation sequencing and phylogenetic signal of complete mitochondrial genomes for resolving the evolutionary history of leaf-nosed bats (Phyllostomidae). *Mol Phylogenet Evol.* 2013;69(3):728–39. 10.1016/j.ympev.2013.07.003 23850499

[ref-8] Challis R Richards E Rajan J : BlobToolKit - Interactive quality assessment of genome assemblies. *G3 (Bethesda).* 2020;10(4):1361–1374. 10.1534/g3.119.400908 32071071 PMC7144090

[ref-55] Cheng H Concepcion GT Feng X : Haplotype-resolved de novo assembly using phased assembly graphs with hifiasm. *Nat Methods.* 2021;18(2):170–175. 10.1038/s41592-020-01056-5 33526886 PMC7961889

[ref-9] Cirranello A Simmons NB Solari S : Morphological diagnoses of higher-level phyllostomid taxa (Chiroptera: Phyllostomidae). *Acta Chiropterologica.* 2016;18(1):39–71. 10.3161/15081109ACC2016.18.1.002

[ref-10] Davis WB : Review of the large fruit-eating bats of the *Artibeus* "lituratus" complex (Chiroptera: Phyllostomidae) in Middle America. Occasional Papers, The Museum, Texas Tech University,1984;93:1–16. Reference Source

[ref-11] de Souza Laurindo R Vizentin-Bugoni J : Diversity of fruits in *Artibeus lituratus* diet in urban and natural habitats in Brazil: a review. *J Trop Ecol.* 2020;36(2):65–71. 10.1017/S0266467419000373

[ref-29] dos Reis NR Peracchi AL : Quiropteros da regiao de Manaus, Amazonas, Brasil (Mammalia, Chiroptera). *Bol Mus Para Emilio Goeldi, Ser Zool.* 1987;3:161–182. Reference Source

[ref-12] Emmons LH Feer F : Neotropical rainforest mammals: a field guide.2nd ed. Chicago, IL: University of Chicago Press,1997. Reference Source

[ref-13] Guerrero JA Ortega J González D : Molecular phylogenetics and taxonomy of the fruit-eating bats of the genus *Artibeus* (Chiroptera: Phyllostomidae). In: C. Lorenzo, E. Espinoza and J. Ortega, eds. *Avances en el estudio de los mamiferos de méxico.*Publicaciones Especiales, México, D.F.: Asociación Mexicana de Mastozoología AC:2008;II:125–146.

[ref-14] Hoofer SR Solari S Larsen PA : Phylogenetics of the fruit-eating bats (Phyllostomidae: Artibeina) inferred from mitochondrial DNA sequences.Occasional Papers, Texas Tech University Museum.2008;277:1–15. Reference Source

[ref-15] Ingala MR Simmons NB Wultsch C : Molecular diet analysis of neotropical bats based on fecal DNA metabarcoding. *Ecol Evol.* 2021;11(12):7474–7491. 10.1002/ece3.7579 34188828 PMC8216975

[ref-16] Kilkenny C Browne WJ Cuthill IC : Improving bioscience research reporting: the ARRIVE guidelines for reporting animal research. *PLoS Biol.* 2010;8(6): e1000412. 10.1371/journal.pbio.1000412 20613859 PMC2893951

[ref-17] Koopman KF : Chiropteran systematics. In: *Handbook of zoology*. *Mammalia.*Berlin: Walter de Gruyter:1994;8(pt 60):1–217. Reference Source

[ref-18] Lariviere D Abueg L Brajuka N : Scalable, accessible and reproducible reference genome assembly and evaluation in galaxy. *Nat Biotechnol.* 2024;42(3):367–370. 10.1038/s41587-023-02100-3 38278971 PMC11462542

[ref-19] Larsen PA Marchán-Rivadeneira MR Baker RJ : Taxonomic status of Andersen’s fruit-eating bat ( *Artibeus jamaicensis aequatorialis*) and revised classification of *Artibeus* (Chiroptera: Phyllostomidae). *Zootaxa.* 2010;2648(1):45–60. 10.11646/zootaxa.2648.1.3

[ref-20] Larsen PA Marchán-Rivadeneira MR Baker RJ : Speciation dynamics of the fruit-eating bats (genus *Artibeus*): with evidence of ecological divergence in Central American populations. In: *Bat evolution, ecology, and conservation.*Springer:2013;315–339. 10.1007/978-1-4614-7397-8_16

[ref-21] Lim BK Engstrom MD Lee TE : Molecular differentiation of large species of fruit-eating bats ( *Artibeus*) and phylogenetic relationships based on the cytochrome b gene. *Acta Chiropterologica.* 2004;6(1):1–16. 10.3161/001.006.0101

[ref-22] Mammal Diversity Database: Mammal Diversity Database. Version 1.13.2024; [Accessed 31 Aug. 2024]. Reference Source

[ref-23] Manni M Berkeley MR Seppey M : BUSCO update: novel and streamlined workflows along with broader and deeper phylogenetic coverage for scoring of eukaryotic, prokaryotic, and viral genomes. *Mol Biol Evol.* 2021;38(10):4647–4654. 10.1093/molbev/msab199 34320186 PMC8476166

[ref-24] Marchán-Rivadeneira MR Larsen PA Phillips CJ : On the association between environmental gradients and skull size variation in the great fruit-eating bat, *Artibeus lituratus* (Chiroptera: Phyllostomidae). *Biological Journal of the Linnean Society.* 2012;105(3):623–634. 10.1111/j.1095-8312.2011.01804.x

[ref-25] Marques-Aguiar SA : A systematic review of the large species of *Artibeus* Leach, 1821 (Mammalia: Chiroptera) with some phylogeographic inferences. *Boletim do Museu Paraense Emílio Goeldi, Série Zoologia.* 1994;10(1):3–83. Reference Source

[ref-26] Marques-Aguiar SA : Genus *Artibeus* Leach, 1821. In: A.L. Gardner, ed. *Mammals of South America*. Chicago, IL: University of Chicago Press,2007;1:301–321.

[ref-27] Morrison DW : Foraging and day-roosting dynamics of canopy fruit bats in Panama. *J Mammal.* 1980;61(1):20–29. 10.2307/1379953

[ref-28] Nurk S Walenz BP Rhie A : HiCanu: accurate assembly of segmental duplications, satellites, and allelic variants from high-fidelity long reads. *Genome Res.* 2020;30(9):1291–1305. 10.1101/gr.263566.120 32801147 PMC7545148

[ref-56] Rao SSP Huntley MH Durand NC : A 3D map of the human genome at kilobase resolution reveals principles of chromatin looping. *Cell.* 2014;159(7):1665–1680. 10.1016/j.cell.2014.11.021 25497547 PMC5635824

[ref-30] Redondo RAF Brina LPS Silva RF : Molecular systematics of the genus *Artibeus* (Chiroptera: Phyllostomidae). *Mol Phylogenet Evol.* 2008;49(1):44–58. 10.1016/j.ympev.2008.07.001 18662791

[ref-50] Reid FA : A field guide to the mammals of Central America and Southeast Mexico (2nd ed.).Oxford University Press,2009. Reference Source

[ref-53] Rojas D Warsi O Dávalos LM : Bats (Chiroptera: Noctilionoidea) challenge a recent origin of extant Neotropical diversity. * Syst Biol.* 2016;65(3):432–448. 10.1093/sysbio/syw011 26865275

[ref-54] Simão FA Waterhouse RM Ioannidis P : BUSCO: Assessing genome assembly and annotation completeness with single-copy orthologs. *Bioinformatics.* 2015;31(19):3210–3212. 10.1093/bioinformatics/btv351 26059717

[ref-31] Simmons NB : Order chiroptera. In: *Mammal species of the world: a taxonomic and geographic reference.*3rd ed.2005;312–529.

[ref-32] Simmons NB Cirranello AL : Bat species of the world: a taxonomic and geographic database.Version 1.6.2024; [Accessed 31 Aug. 2024]. Reference Source

[ref-33] Simmons NB Voss RS : The mammals of Paracou, French Guiana, a Neotropical lowland rainforest fauna. Part 1, Bats.Bulletin of the AMNH.1998;237:1–219. 10.5281/zenodo.4545052

[ref-34] Vurture GW Sedlazeck FJ Nattestad M : GenomeScope: fast reference-free genome profiling from short reads. *Bioinformatics.* 2017;33(14):2202–2204. 10.1093/bioinformatics/btx153 28369201 PMC5870704

[ref-51] Wilson DE : Mammals of the Yucatán Peninsula. In: Mares, M.A., & Schmidly, D.J. (Eds.), *Latin American mammalogy: History, biodiversity, and conservation.* University of Oklahoma Press.1991;143–162.

[ref-57] Zhou C McCarthy SA Durbin R : YaHS: Yet another Hi-C scaffolding tool for genome assembly. *Genome Biol.* 2023;24:68.37024973

[ref-35] Zortea M Mendes SL : Folivory in the big fruit-eating bat, *Artibeus lituratus* (Chiroptera, Phyllostomidae) in eastern Brazil. *J Trop Ecol.* 1993;9(1):117–120. 10.1017/S0266467400007057

