# Supplementary material for: The genome sequence of Artibeus lituratus (Chiroptera, Phyllostomidae, Stenodermatinae; Olfers, 1818)
Source: Wellcome Open Res. 2025 Apr 1;10:170. [Version 1] doi: 10.12688/wellcomeopenres.23088.1 (PMC12344409; doi:10.12688/wellcomeopenres.23088.1)
Supplement: Peer review history

# Reviewer report: Reviewer response for version 1

**DOI:** 10.21956/wellcomeopenres.25424.r127425

**Published:** 2025-08-12

**Authors:** Gabriela Castellanos-Morales

**Affiliations:** 1 El Colegio de la Frontera Sur, Tabasco, Mexico

Simmons et al. present the genome assembly for Artibeus lituratus a North American frugivorous bat from family Phyllosotmidae. The phylogenetic postition and morphological characteristics of the species are described. The report of this genome was performed as part of the Bat1K project. The authors obtained HiFi reads and Hi-C data to get a chromosome level genome assembly. The assembly was carried out following the Vertebrate Genome Project Galaxy pipeline v2.0. Assembly and raw data are avaliable at NCBI. The assembly has good quality.

 I recommend providing more detail about the assembly pipeline as not all readers will be familiar with the Vertabrate Genome Project Galaxy pipeline. Please provide the values for the parameters used in the pipeline; for example mention the kmer size used at the begining of the kmer profiling step, as different values will give different results. Also, was the name of the restriction enzymes used in the Hi-C scaffolding used in the scaffolding step of the workflow?

Are sufficient details of methods and materials provided to allow replication by others?

Partly

Is the rationale for creating the dataset(s) clearly described?

Yes

Are the datasets clearly presented in a useable and accessible format?

Yes

Are the protocols appropriate and is the work technically sound?

Yes

Reviewer Expertise:

Population genomics and biodiversity conservation

I confirm that I have read this submission and believe that I have an appropriate level of expertise to confirm that it is of an acceptable scientific standard.

# Reviewer report: Reviewer response for version 1

**DOI:** 10.21956/wellcomeopenres.25424.r121802

**Published:** 2025-04-11

**Authors:** Jamie McGowan

**Affiliations:** 1 Earlham Institute, Norwich, UK

This Data Note from Simmons et al. reports the genome sequence of the great fruit-eating bat *Artibeus lituratus*.

 PacBio HiFi reads and Illumina Hi-C reads were generated from an individual male bat. The genome assembly was constructed with the VGP Galaxy pipeline using Hifiasm for assembly and YaHS for scaffolding. The reported assembly is very high quality with a scaffold N50 of 160 Mb, 97.7% of sequence assigned to chromosomal-level scaffolds, and a BUSCO completeness score of 98.2%. The authors generated a phased assembly and have deposited both haplotypes, although the manuscript only reports on one haplotype assembly.

 Overall, this Data Note is well written. The raw sequencing data and assemblies have been deposited, and the methods are sufficiently detailed to enable reproducibility.

**I only have minor comments that the authors may wish to address:**

1. It would be informative to mention the expected karyotype (2n=31 male) and sex chromosome system (XY1Y2 male) in the introduction. There are several published cytogenetics studies of *A. lituratus *that could be referenced.
2. Table 1 has two * notes mentioning BUSCO datasets and version info. However, Table 1 does not contain the BUSCO results.
3. Typo/missing bracket: “reads (contig N50 [70 Mb) was generated after removal”

Are sufficient details of methods and materials provided to allow replication by others?

Yes

Is the rationale for creating the dataset(s) clearly described?

Yes

Are the datasets clearly presented in a useable and accessible format?

Yes

Are the protocols appropriate and is the work technically sound?

Yes

Reviewer Expertise:

genomics

I confirm that I have read this submission and believe that I have an appropriate level of expertise to confirm that it is of an acceptable scientific standard.
